# Innovation Pathways in the NHS: An Introductory Review

**DOI:** 10.1007/s43441-021-00304-w

**Published:** 2021-05-19

**Authors:** Anmol Arora, Andrew Wright, Tsz Kin Mark Cheng, Zahra Khwaja, Matthew Seah

**Affiliations:** 1 School of Clinical Medicine, University of Cambridge, Addenbrooke’s Hospital, Cambridge, CB2 0SP UK; 2 Department of Physiology, Development and Neuroscience, University of Cambridge, Cambridge, UK; 3 Department of Medical Sciences, Faculty of Biology, University of Cambridge, Cambridge, UK; 4 Department of Pathology, University of Cambridge, Cambridge, UK; 5 Department of Surgery, University of Cambridge, Cambridge, UK

**Keywords:** Entrepreneurship, Investment, Technology, Quality, Safety

## Abstract

Healthcare as an industry is recognised as one of the most innovative. Despite heavy regulation, there is substantial scope for new technologies and care models to not only boost patient outcomes but to do so at reduced cost to healthcare systems and consumers. Promoting innovation within national health systems such as the National Health Service (NHS) in the United Kingdom (UK) has been set as a key target for health care professionals and policy makers. However, while the UK has a world-class biomedical research industry, several reports in the last twenty years have highlighted the difficulties faced by the NHS in encouraging and adopting innovations, with the journey from idea to implementation of health technology often taking years and being very expensive, with a high failure rate. This has led to the establishment of several innovation pathways within and around the NHS, to encourage the invention, development and implementation of cost-effective technologies that improve health care delivery. These pathways span local, regional and national health infrastructure. They operate at different stages of the innovation pipeline, with their scope and work defined by location, technology area or industry sector, based on the specific problem identified when they were set up. In this introductory review, we outline each of the major innovation pathways operating at local, regional and national levels across the NHS, including their history, governance, operating procedures and areas of expertise. The extent to which innovation pathways address current challenges faced by innovators is discussed, as well as areas for improvement and future study.

## Introduction

The industry of healthcare is recognised as one of the most innovative, with a record of driving multidisciplinary innovation and fostering the adoption of new technologies. Health innovations tend to drive improvements in patient care as well as making this care increasingly cost-effective. This is of particular interest to healthcare systems such as the National Health Service (NHS) in the United Kingdom (UK), which seek to deliver high-quality care under tight budget constraints. The UK government’s spending on healthcare is approximately 10% of gross domestic product (GDP) and an ageing population coupled with increases in long-term health conditions may only increase this in the foreseeable future [1]. The moral imperative towards improvements in patient care, and the need for these improvements to be cost-effective, means that facilitating innovation within and around health systems such as the NHS is a key priority for policy makers and clinical leaders. Innovation in healthcare systems such as the NHS, is partly driven by intrapreneurship whereby members of the organisation pursue innovative ideas, but is also largely propelled by entrepreneurs creating ideas and partnering with healthcare partners. The NHS Long Term Plan, published in 2019, highlighted the importance of innovation in the NHS, including the role it has in improving health outcomes, and committed the NHS to developing the infrastructure required for innovations to thrive [2]. It emphasised genomics as an innovation with potential to benefit both children and adults with rare diseases and/or cancer. Mental health was also highlighted as an area for future innovation efforts particularly in regards to patient reported outcomes.

It has been noted that only a tiny proportion of medicines and medical devices which reach the market succeed and that the journey to market can sometimes cost in the billions of pounds and take over a decade [3]. In recent years, there have been numerous innovation pathways made accessible to early-stage innovators seeking to incorporate new technologies, services or digital innovations in the NHS. The introduction of these pathways has formed part of the dual strategy of improving health and creating wealth within an expanded British Life Sciences industry, led by the Department of Health and Social Care and the Department for Business, Energy and Industrial Strategy [4–6].

Two key stages in an innovation process are invention and implementation, with an intervening development stage [7]. The invention stage is largely the responsibility of the 'inventor(s)'; it consists of formulating the idea for an innovation and developing the service or product. By the time the innovator approaches an innovation pathway, the invention stage would normally be resolved. However, it does present a substantial barrier to entry in many cases due to resources needed to develop a viable product. The implementation stage, on the other hand, can be even more resource intensive, expensive and arduous. Since this stage consists of testing the innovation and incorporating it in health systems, access to patients is typically required. Diffusion of innovation is well characterised by the ‘Diffusion of Innovations’ theory proposed by Everett Rogers [8]. The theory dictates that the rate of adoption of an innovation is initially slow until it reaches a critical mass, after which spread of the innovation is self-sustaining. Therefore, the early period of the implementation stage can be particularly challenging for innovators to navigate, both because of the resources that are required and the need to identify potential early adopters.

It is in this implementation stage that innovation pathways may offer the greatest assistance to innovators. Fundamentally, these innovation pathways (or programmes) would help early-stage innovators find markets for their products. Additionally, they may help them with clinical testing and provide some funding. In some cases, they are comparable to the services that accelerators or venture capital firms might provide to early-stage start-ups but they also provide specialist support to overcome healthcare-specific barriers to entry, such as the need for testing to ensure patient safety. In addition, many innovation pathways focus on forming a network between different stakeholders, simplifying aspects of the innovation pipeline.

These pathways support a wide range of innovations, ranging from medical devices to the development of new therapies. In many cases, the pathways are not specific to a certain type of innovation though there are some which have funding streams dedicated to certain avenues. The advent of industry 4.0 and machine learning, as well as the synergies between different innovations, require that pathways be increasingly open given that the line between hardware and software innovations is increasingly blurred.

This introductory review seeks to offer an overview of innovation pathways available within and around the NHS with a view to provide insight to innovators and map out potential routes to market entry. In doing so, the review also seeks to illustrate any overlap or deficiencies in the current system.

## Local Pathways

For innovations which are designed to impact patient care, a key component of their development is the need to test products and services in clinical environments. Local innovation pathways integrated in hospital trusts permit this and offer resources to innovators seeking to implement their devices or services in the NHS. In local systems, there are numerous stakeholders, including clinicians, patients, hospital trusts and Clinical Commissioning Groups (CCGs). CCGs plan health services in their local area and they are responsible for two-thirds of the total NHS budget [9]. They work closely with hospitals and other healthcare providers in their local area to organise care pathways and may also collaborate with Academic Health Science Networks (AHSNs; see below). The 2021 NHS White Paper set out further integration of local health and care, in the form of Integrated Care Systems (ICSs), which will succeed CCGs [10]. It is yet unclear how these changes will affect the innovation role of local health systems.

Individual trusts may offer their own services to innovators, such as through the Test Beds programme. The Test Beds programme is a cross-government venture funded centrally to provide hospitals with the resources to support innovations at a local level [11]. Evidence is generated and evaluated to explore potential for wider implementation within the NHS.

### NHS Vanguard

NHS Vanguard test sites are a similar venture to test beds, 50 of which were selected in 2015 to propel development of innovative care models which could then be applied nationally [12]. Vanguard sites consist of a group of stakeholders in a local area working together to test, measure and evaluate innovative care models. Though Vanguard sites are local pilots they receive national support and are intended to lead innovation at a national level. Vanguard sites operate in a range of fields, including general practice, acute care and care homes [13].

### Academic Health Sciences Centres

Academic Health Sciences Centres (AHSCs) are accredited institutions in the UK which are recognised for their research and clinical expertise. They typically consist of a partnership between a teaching hospital and a university. AHSCs have been established in North America for decades, but have expanded internationally and have been recognised in the United Kingdom since 2007 with the launch of Imperial College London’s AHSC [14]. AHSCs permit collaboration locally among research institutions and care providers as well as globally between international AHSCs. It has been noted that AHSC partnerships between universities and hospitals may work to streamline approvals for clinical research by combining research infrastructure, for example “Joint Research Offices” [14].

## Regional Pathways

As the 2018 RAND Europe review of UK Healthcare identified [15], there is a pressing need in the NHS innovation landscape for national policy which supports regional innovation, and for regional success to then shape national policy and implementation. A similar point was emphasised in the 2021 Health and Care White Paper [10]. Therefore, it is important to consider not only the local and national infrastructure for innovation, but also regional networks.

### Academic Health Science Networks

**Fig. 1.**
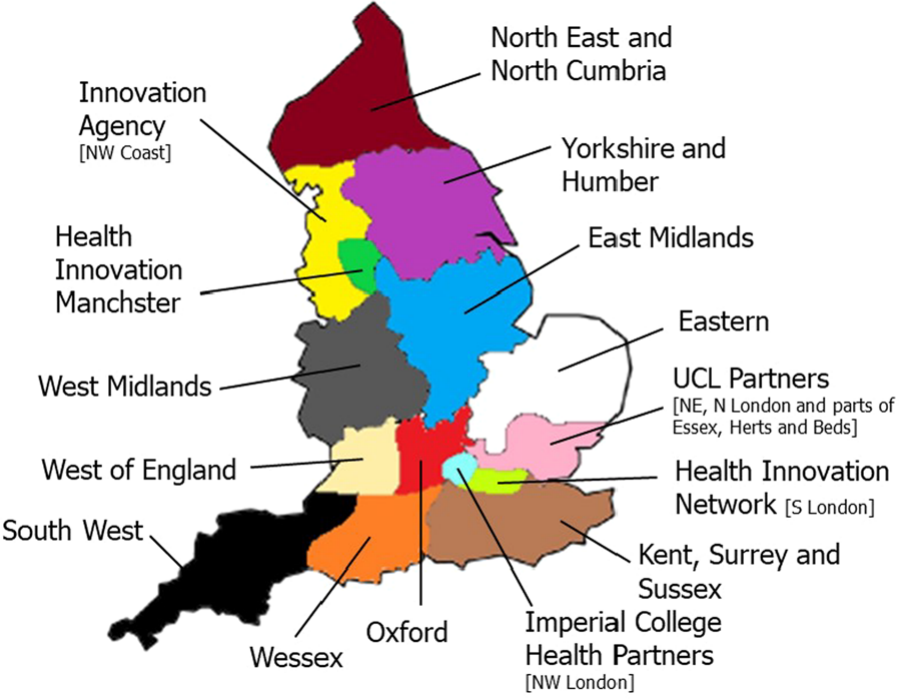
Area map of AHSNs.

Perhaps the most important regional infrastructure for innovation is the network of AHSNs [16, 17]. This network was established in 2013 following a series of policy and academic reports [18], recommending significant reform in the innovation landscape of England [4, 19] with the aim of improving regional health and creating wealth. AHSNs comprise 15 regional bodies (see Fig. 1), overseen by NHS England, which aim to coordinate regional NHS trusts, local government, charities and industry, helping to identify and spread health innovations [5, 16]. According to a 2016 report compiled by the Office for Life Sciences and Monitor Deloitte [20], AHSNs represent the only body, regional or national, that has competency across every stage of innovation, from idea to implementation. Their 2018/2019 report [17] contains more detail on the impact of AHSNs, including work on 3,630 innovations, creating 691 jobs and leveraging over £150million of investment.

The regional nature of AHSNs is important because they are well placed to take advantage of individual academic and industrial strengths of regions, using a range of strategies such as innovation scouts [21] and close partnership with university research hubs such as AHSCs [22] to identify and implement innovations. Examples include Digital Health London [23] delivered by MedCity, London’s AHSNs and AHSCs to develop the capital’s digital health infrastructure. Conversely, the universal coverage across England means that AHSNs can operate as a network to deliver national policy objectives. This includes both central programmes set at the beginning of each financial year [24] and unexpected challenges, for example implementing digital primary care in the COVID-19 crisis [25]. The first years of the AHSN network have widely been regarded as a success, and in 2017 NHS England relicensed the networks, with an increase in funding [26]. There are still challenges to their operation, however. The only current full review of their performance, which covered their operation up to 2016, identified significant variation in the practice of AHSNs [18]; some work on a looser basis, working with a wider range of companies and institutions, while others work more tightly with fewer institutions. This was partly due to pre-existing NHS structures, such as AHSCs and Collaborations for Leadership in Applied Health Research and Care (CLARHCs, now called ARCs, see below).

### Applied Research Collaborations

Applied Research Collaborations (ARCs) are regional programmes funded by the National Institute of Health Research (NIHR). They replaced the CLAHRCs in 2019 [27]. They are local partnerships between AHSNs, NHS, universities and charities; their 15 regions overlap almost directly with the regions of the AHSNs. From 2019, £135 million will be invested, with the specific aim of increasing the rate at which innovative research is adopted in clinical practice. Each ARC takes a lead on a specific area based on the expertise of their region, with priority areas identified from RAND Europe’s ‘Future of Health (2017)’ [28] review. The Wessex ARC, for example, has a particular focus on ageing and dementia. Therefore, while the ARC remit is outside the traditional NHS innovation framework, they are a key piece within the regional innovation landscape; UCLPartners (North London AHSN) and the North Thames ARC, for example, have collaborated on a project investigating the long-term physical impacts of COVID-19 [29].

### Regional Medicines Optimisation Committees

Regional Medicines Optimisation Committees (RMOCs) were established in 2016 to optimise the use of medicines across the NHS. Overseen by NHS England, they bring together decision makers, clinicians and patients. According to their 2019 operating model [30], their role is to share clinical best practice, speed implementation of new medicines, instigate changes in use when evidence base changes, and reduce unwarranted variation in prescribing. There are currently four RMOCs covering England (South, London, Midlands and East, North), but there are plans to expand this number to seven from Autumn 2020. Similar to the ARCs, each RMOC takes a lead on a specific priority area (with these areas decided by NHS England Medicines Value Programme). For example, the London RMOC leads work on polypharmacy [31]. Each RMOC is also able to propose its own priorities, suggested by local clinical care groups and area prescribing committees, which are then presented to the national priorities panel for consideration. Similar to the AHSN network, this facilitates top-down and bottom-up consideration of innovation priorities and policy changes.

## National Pathways

### Accelerated Access Collaborative

The Accelerated Access Collaborative (AAC), established in May 2019, is the umbrella body across the UK health innovation ecosystem [32]. It was founded following the Accelerated Access Review led by Lord Darzi, which highlighted major areas of improvement for NHS Innovation [33]. The unit operates within NHS England and NHS Improvement, with board members representing each of its key partners [34]. With immediate goals of creating a “single front door to the innovation ecosystem”, including an online portal with information, and support and signposting for innovations, the AAC strives to guide teams across the entire innovation pipeline, ranging from local testing provided by AHSNs, or evidence queries related to National Institute for Health and Care Excellence (NICE) submissions [35].

Since June 2019, the AAC have focussed on providing support for three categories of early-stage products (those yet to be approved by NICE) [36]:

- Advanced Therapy Medicinal Products
- Histology Independent Treatments for Cancer
- Artificial Intelligence

The AAC runs and coordinates a number of programmes across the UK innovation ecosystem. Some of these involve existing initiatives (such as AHSNs), some are dedicated AAC programmes such as the Clinical Entrepreneur Training Programme, where AAC provides training to help clinicians bring innovations to market [37], and some programmes run with other services, such as the NIHR Artificial Intelligence (AI) Scheme [38].

### NHS Digital

Previously known as the Health and Social Care Information Centre [39], NHS Digital is a public-facing body [40], providing most web-services, statistical publications, and data management systems used within the NHS [41]. Their focus is largely centred on the current infrastructure of the NHS, and thus they tend to have a restricted role in early product development; rather, they innovate through continuous improvement of existing services, including NHS websites and the NHS app. That being said, NHS Digital has a specific drive for better data collection and easier patient–clinician interactions. With more than 700,000 users registered to the NHS app at the end of May 2020 [42], combined with over 90 million demographic records on the NHS Spine system [43], NHS Digital is an essential component of the innovation pathway, particularly in the evidence generation phase. One example of this is the contribution of NHS Digital to the upcoming AI award, by producing synthetic datasets with the Medicines and Healthcare Products Regulatory Agency (MHRA) to optimise accuracy of algorithms [44].

### NHSX

In comparison to NHS Digital’s more administrative role, NHSX reports directly to the Department for Health and Social Care and the Chief Executive of NHS England. They act as the oversight organisation to define the digital strategy envisioned by the Health Secretary [45], and other long-term strategies, including the future remit of NHS Digital [46]. A specific focus of NHSX is to accelerate digitisation within the NHS by establishing centrally agreed standards and allowing local NHS organisations the freedom of choosing their mode of delivery, if they meet the required open standards for interoperability, accessibility, and security [45]. They also prioritise cutting time spent by clinicians inputting and accessing data within the NHS system, ease of access to key NHS services by patients on their smartphone and ensuring secure and reliable access to essential diagnostic information in clinic [47].

The aim of NHSX to establish a data-driven ecosystem will not only allow patients to have easier and wider access to their personal data, but also easier circulation of the collected data between patients, clinicians, and care systems. The increase in interoperability between different health institutes, by better data sharing practices including cloud storage, has the potential to improve patients’ access to services, deliver the right diagnostic information to clinicians, and provide researchers with the healthcare data they need [48]. It would also provide an innovator friendly environment for easier product testing and initial health system adoptions. Furthermore, it assists in the goal of the AAC of achieving proper ‘demand signalling’, whereby researchers, innovators and funders can more easily understand what the NHS needs [49].

### NIHR Funding Schemes

National Institute for Health Research (NIHR) is a UK government agency which funds research into health and care. It has a wide variety of programmes which support different groups of research, spanning the research innovation pathway from early Minimal Viable Product development to evaluative research, including pragmatic clinical trials. NIHR schemes undergo regular mandated peer review, to ensure appropriate design and methodology.

The NIHR runs many schemes, some of which are specifically aimed at innovators [50], including the competitive i4i (Invention for Innovation) award [51]. This has a track record of highly successful adoption and commercialisation, although less than a quarter of applicants are successful per year [52]. Life sciences companies can apply to most programmes as lead applicants, or in some cases, as co-applicants alongside industry, NHS and academic partners. The i4i programme is a translational funding scheme, aimed at advancing medical technologies and interventions, especially in areas of high or rapidly increasing demand. In 2020 this included the themed call of injuries, accidents, and urgent and emergency care [53] and more recently, the COVID-19 call [54]. It is split into three awards, Product Development Award (PDA), Challenge, and Connect, aimed at different stages of development.

With the goals of minimising investment risk and assisting development of translational innovation into real ideas, the awards support both existing ideas, with research funding used to reach the next stage in the developmental pathway, and additional research for newly, CE (certification) marked products, where regulators such as NICE require more evidence before adoption. All awards are aimed at products and services which aim to integrate into the NHS [55].

While the Challenge award supports assessment of medical innovations in the real-world, the PDA provides all justifiable expenses for translational research proposals [51]. Connect, on the other hand, is a programme centred on small and medium enterprises, focussing on helping early-stage products to build momentum and reach important business and technological milestones [51].

### NIHR Artificial Intelligence Scheme

In August 2019, NHSX launched an AI Lab, using a £250 million fund from the Department of Health and Social Care [56, 57]. £140 million of this has been made available over three years in the Artificial Intelligence in Health and Care Award [58], as a collaboration between NHSX, the AAC, and the NIHR. The aim of this award is to advance the development of AI technologies which meet the strategic aims of supporting data-driven decision making and optimising interactions between existing systems, as outlined in the NHS Long Term Plan [48]. The funding focuses on four NHS priority areas: screening, diagnosis, decision support and improving system efficiency [58]. Support is available across four phases of development (which are similar to the phases of drug development): Phases 1–3 are incorporated into NIHR’s i4i and SBRI (Small Business Research Initiative) award schemes, while Phase 4 is covered by NHSX via the AAC team. A call for applicants is made twice per year. The award provides awardees assistance in navigating issues in the developmental process, highlighted by NHSX’s recent AI report [49] and digital innovator summary report [59]. These issues include the vital process of patient data access.

NHSX also runs a number of other projects to drive AI innovation in the NHS, under the umbrella of the NHS AI Lab. In 2020, the NHS AI Lab announced its Skunkworks project to find, fund and resource AI endeavours within the health and care ecosystem [60]. NHS colleagues can pitch problems with potential AI solutions in pitching-style events to identify real-life problems which can benefit from the expertise of the NHS AI Skunkworks team.

### NHS Innovation Accelerator

Launched in 2015, the NHS Innovation Accelerator is an NHS England initiative delivered in partnership with England’s 15 AHSNs and hosted at UCLPartners [61]. The Innovation Accelerator invites applications from individuals (clinical, industry, academia) as part of an annual international call. Applicants are required to demonstrate their skills and experience to qualify for support, alongside the efficacy and safety of the proposed innovation, as well as a strategy for scaling in the NHS [61]. The assessment panel is drawn from a wide range of organisations including NHS England and NHS Improvement, AHSNs, NICE and The Health Foundation. Successful Innovation Accelerator fellows receive bespoke support, including access to mentorship from a range of high-profile experts, links with AHSNs and other stakeholder organisations, peer-to-peer learning and support, a dedicated learning programme, and a bursary. Innovations can be of any type, including medical devices, apps, new models of care and artificial intelligence [62]. While the NHS Innovation Accelerator core team provides day-to-day support, AHSN partners provide signposting, local networking and support on scaling strategies.

The Innovation Accelerator has supported a number of successful innovative solutions during the COVID-19 pandemic, both in response to new demands and exacerbations of existing problems. Examples include ‘Echo’, an app allowing patients to order NHS prescriptions to their home for free, and ‘HaMpton’, which uses an app for home monitoring blood pressure during pregnancy [63].

### Innovate UK

Innovate UK (formerly known as Technology Strategy Board) provides grants to companies that are working on projects that will develop something that can be classed as a “technology innovation”. Innovate UK is part of UK Research and Innovation (UKRI), and supports innovative ideas and business growth through grant funding, loans and procurement [64].

Innovate UK aims to invest in early-stage innovation projects with high potential as well as focussing on sectors it has identified as priorities. Each funding opportunity has different eligibility requirements but in general, eligible projects are divided into four broad Research and Development (R&D) activities [65]:

- Fundamental research
- Feasibility studies
- Industrial research
- Experimental development

Innovate UK is not a health-specific pathway, but they do operate ‘catapult’ innovation centres, which focus around innovation in specific areas of health, such as the Medicines Discovery Catapult [64]. They have collaborated with a range of other partners, including the charity LifeArc, the Multiple Sclerosis (MS) Society and The University of Manchester [66]. The Medicines Discovery Catapult has been particularly active during the COVID-19 pandemic, including through the coordination of the UK Lighthouse Labs Network and in providing leadership to the UK’s drug discovery community [67].

### Medilink UK

Medilink UK is a not-for-profit, professional association and specialist health and life-science consultancy. Its primary focus is on fast tracking the development of Life Science companies by enhancing their connectivity to UK business, clinical, regulatory and finance communities, helping form new partnerships and navigate the increasingly complex health innovation landscape. Other services offered include market research, PR/communications, assistance with market access (including international) and assistance with grant applications [68].

One of the benefits of Medilink to small and medium enterprises is the access to the NHS. Medlink has links with the regional AHSNs, and they also help companies with NHS market access strategy. For example, their *Innovation Surgeries* programme starts with an offer to all regional companies, but especially small and medium enterprises who have a product or service that they believe should sell to the NHS and is either close to or on the market but struggling to find buyers. Certain companies may also participate in the aforementioned regional ‘Test Beds’ programme in collaboration with NHS trusts, CCGs and clinical trials units [69]. An example of a successful venture supported by Medilink UK is Deltex Medical, a company who produce the CardioQ-ODMOesophageal Doppler Monitor (ODM), a minimally invasive Doppler probe that can be inserted nasally or orally to measure circulating blood volume [70].

### NHS Clinical Entrepreneur Programme

The NHS Clinical Entrepreneur Training Programme launched in 2016 as an entrepreneurial workforce development programme to equip participants with the skills, knowledge and experience required to develop innovations within the NHS [37]. Since the inception of the programme, it has trained over 500 healthcare professionals with mentoring, networking opportunities and teaching [37]. The programme is available to clinical and non-clinical NHS staff seeking to develop an idea for an innovation which may improve patient care. Such a programme is important in fostering a culture of improvement within the NHS, helping to encourage intrapreneurship and reducing attrition rates of staff leaving to pursue entrepreneurial aspirations.

## Alternative Pathways

For those seeking direct commercialisation, there are several other options to support innovations that exist outside normal NHS and government structures. Funding can be provided by angel investors, venture capitalists and family offices. Start-up accelerators provide capital too, often combined with close mentorship and upskilling of the team. Any venture can seek this type of funding, and the process involves a business pitch to the investors in question and often a series of interviews with the start-up team. There is no formal process for obtaining funding from venture capitalists and Angels and the process usually involves cold-communication and networking.

### Angel Investors

Angel investors are high net worth individuals who invest into the early formation of a start-up. In return for the provision of funding, angels usually get a stake in the company. Many angels have specific areas of expertise, so they can advise and help build the connections of the venture—this is because angels have a personal responsibility for the start-up’s success. Angels are attractive sources of capital since they afford more flexibility than investment banks (for example) and usually their loan does not have to be paid back if the venture fails. Angels are usually part of a larger network and intermediary agents such as MedCity (the umbrella organisation for London’s AHSCs and several universities [71]) work to connect them to life-science start-ups. An example of an established angel network is Cambridge Angels [72], while examples of life-science start-ups showing success from such investment include Eagle Genomics and Smart Target [73].

### Venture Capital

Venture capital is a type of financing invested in start-ups that are usually high risk but have the potential for rapid growth. The investments are usually larger than angels and so the venture should ideally have the management and strategic plan to scale quickly. Different venture capitalists operate at different stages in the venture’s life-cycle (from idea to adoption) [74]. The clear benefit for businesses is the lack of obligation to pay back lent money. Like angels, venture capitalists have business and institutional knowledge and are well connected, and again like angels, venture capitalists expect a return for their investment, usually through an acquisition or access to intellectual property. Many start-ups are wary of very large investments as they may risk losing management of the company. The oldest venture capitalist in the UK is Abingworth, which boasts a successful portfolio and series of exits [75].

### Start-up Accelerators/Incubators

Start-up accelerators and incubators provide intense training, upskilling and mentorship for select ventures, alongside funding. This is particularly important for early-stage spin-outs (often projects started in university research groups) as it ‘accelerates’ the acquisition of strategic, managerial and commercial acumen. Some pre/seed stage venture capitalists and angels can offer similar help but this is not in the form of a structured programme such as these accelerators. Many are located at life-science venture ‘hot-spots’ such as Cambridge, United Kingdom. An excellent example is Start Codon [76], which leverages its connections with the biomedical hub in Cambridge to provide seed funding and training for innovators, particularly those taking an idea from academic research into a start-up, in return for an 8% stake in the final product [77].

### Equity Crowdfunding

Crowdfunding is a fairly new model for ventures to secure capital. It allows life-science start-ups raise up to £1 m (and sometimes more) by attracting a small pool of investors. Equity crowdfunding gives more control to the entrepreneurs in terms of later funding and strategic decisions since the capital has been acquired by many individuals rather than one body as in venture capital and angel investing. A great example of a body is Capital Cell and a recent success story is the French Antabio [78].

### Healthcare Leadership Academy

Healthcare Leadership Academy is a social enterprise run privately by medical professionals aimed at providing skill-based support for medics entering the entrepreneurship arena. It has teaching fellows from across the UK clinical, academic and health policy establishment. Similar to the AAC Clinical Entrepreneur Training Programme, it provides training rather than funding, although it operates independently of the NHS [79].

## Discussion

**Fig. 2.**
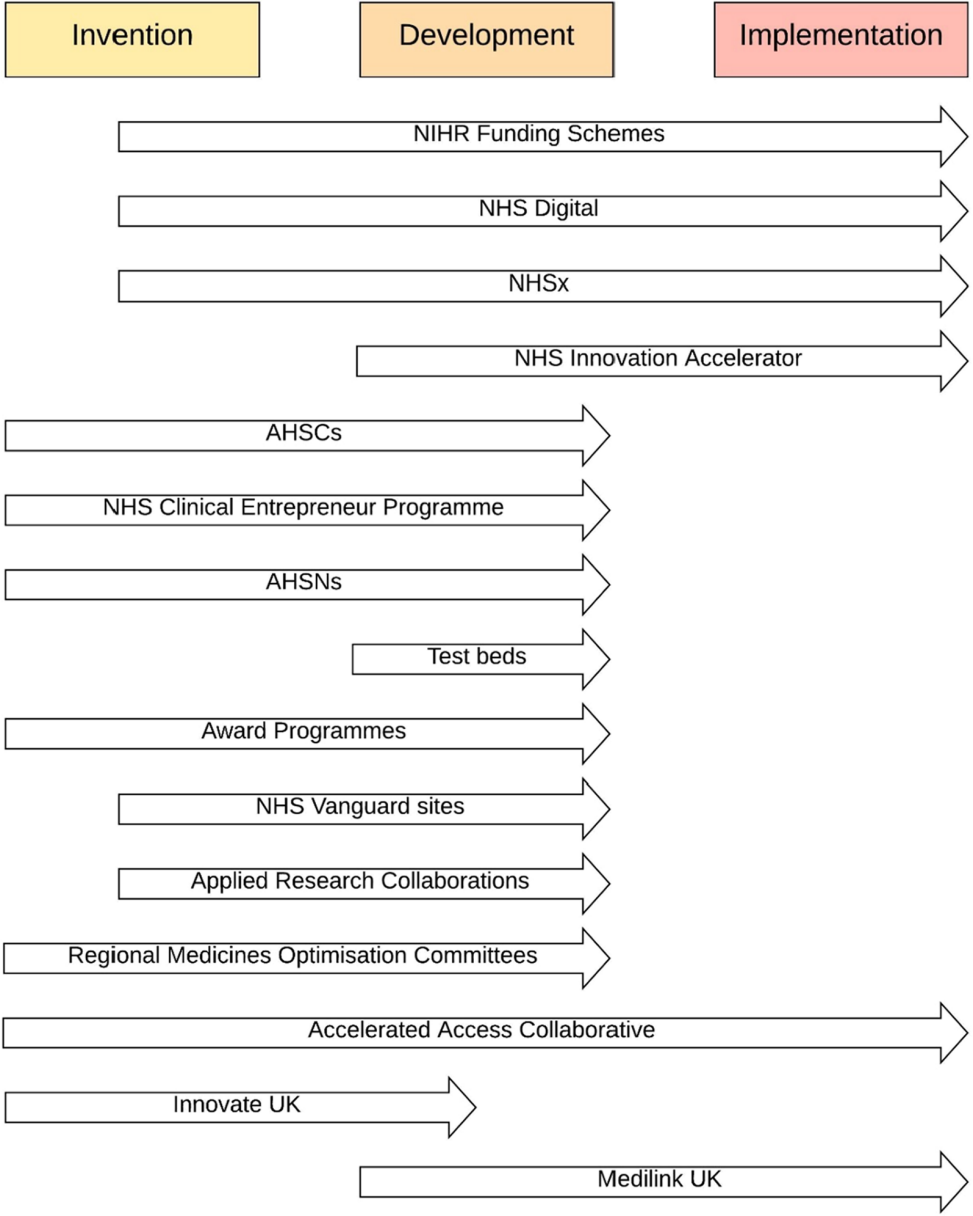
Innovation pathways in the context of the conventional innovation pipeline.

The goal of making the healthcare sector more efficient and effective in all three stages of innovation (invention, development and implementation) is a primary concern for health companies and governments around the world [80]. There has been significant emphasis on improving the development and adoption of innovations in the UK since the 2000s, when a series of reports identified significant deficits in healthcare innovation in the NHS [4, 5, 19]. Since then, several pathways have been set up, operating across all stages of the innovation pipeline (Fig. 2), with their scope defined by location, technology area or stage of development, based on the specific problem identified when they were set up.

Despite this strong policy push, there remain significant barriers to achieving the political ambition of making the NHS the most innovative health system in the world [81]. While one might expect that national policy would be more easily implemented in a relatively unified health system such as the NHS, rather than more fragmented structures, in many areas the UK still lags behind. For example, the UK public are much less likely to use digital health services to track medical conditions compared to European nations, suggesting relatively low integration of digital health within the NHS compared to other health systems [82]. While academic literature exploring the current innovations pathways is limited, there have been efforts to evaluate the uptake and diffusion of innovation previously, perhaps most notably in the 2012 Innovation, Health and Wealth report, which recommended implementation of AHSNs to align innovation with healthcare delivery and improve patient outcomes [83]. Since then, it has been suggested that budget silos and a lack of accountability for innovation may be hindering greater success of innovation pathways such as the AHSNs [18, 84, 85].

While the body of this paper has focussed on individual pathways, the following discussion will cover the major challenges for innovation in healthcare, and the extent to which the discussed pathways have addressed these challenges.

### Challenges to Implementation: Connecting Stakeholders, NHS Bureaucracy and Interoperability

It is widely recognised that while the NHS, and the UK Life Sciences industry more generally, is world leading in the invention and early development of medical devices [86], there are significant barriers to implementing these innovations [4]. One reason for this is the fragmented nature of the NHS, which presents a barrier particularly for small and medium enterprises who may be unaware of impactful regional variations, from digital infrastructure to bureaucratic structures. For example, companies seeking to introduce digital health technology into NHS services will likely gain traction in urban areas such as Greater Manchester and London/South East, but might struggle much more in other parts of the country where primary care remains stubbornly paper-based [86].This may explain why informal clinical networks, long identified as a key component of innovation adoption [87], provided by the AHSNs and other regional/local innovation pathways have been so successful. They can guide innovators through the particular landscape of their area, helping them overcome many of the traditional barriers to implementation, from market access to ensuring the continued use and improvement of innovations [18]. A recent example is the work of Oxford AHSN in aiding a personalised oncology company through the process of product testing and market access [88]. The need for regionalisation in delivering health services is exemplified in the initial problems faced by the national Track and Trace system for COVID-19 [89, 90]. Further regional and local integration will likely arise from the establishment of ICSs as the future of health and care delivery in England, as announced in 2021 [10]. It is not yet clear how the new integrative ICSs will affect the local and regional economy of innovation, particularly given the other measures to centralise power in the NHS set out in the white paper, but it is expected that they will improve IT integration and enhance the sharing of data. Of course, the aim of interoperability within the NHS (the ability of NHS digital systems to talk to each other) [91], which is a major goal of NHSX [92], will allow improvements in local integration to be scaled up to national coordination of care, aligning with the goal set out in the NHS Long Term Plan to streamline and accelerate the innovation pipeline [2].

A remaining challenge for implementation lies in the overlapping nature of the innovation pathways, which could be described as being disjointed. This is in part due to the patch-work manner in which they were set up. While the recent establishment of the AAC as the parent body of the NHS innovation landscape is a step in the right direction, providing a ‘front door’ to the confusing landscape of pathways, there remains a lot of work to do. The focus of the AAC on ‘demand signalling’, working out what innovation priorities the NHS has, is vital, and it is key that this receives both top-down and bottom-up (from CCGs and AHSNs) input [18]. This approach has already proved successful for specific services such as the RMOCs, where local bodies are able to suggest specific priorities for the region which are then approved by a national body; this takes into account both regional needs and national priorities [30]. A key role of the AAC must therefore be the identification of models of best practice, for wider dissemination through the innovation landscape.

### Challenges to Development: Prototyping and Testing

While a particular problem for NHS innovation is the confusing process of implementation, a general issue for healthcare innovation is the process of development. Ranging from early clinical testing through to regulatory approval, the necessary safety and quality checks can represent a substantial barrier for innovators lacking connections and funds [93]. In this regard, local pathways including the NHS Vanguards and CCG Test Beds schemes have provided an invaluable asset to coordinate testing of innovative MedTech, digital health solutions and care models. It is important to note the NHS Long Term plan discusses plans to expand the Tests Beds scheme through to Regional Test Bed Clusters, with an increasing share of the NHS funding spent on real-world testing [2]. Similarly, the introduction of ICSs will further integration of health and social care, potentially further facilitating real-world evidence generation [10, 94]. At a regional level, the ARCs and AHSNs once again provide a crucial link across industry, NHS and academic stakeholders, while at a national level schemes such as the NHS Innovation Accelerator and Medilink allow easier access to the AHSN network. This is particularly key when it comes to generating evidence for the marketing of a product, pre-implementation across the NHS, and for gaining regulatory approval from EU and UK bodies and final adoption hurdle of HTA (Health Technology Assessment, which in England is conducted by NICE), the process of which is often very opaque [93].

### Challenges to Development: Accessing Clinical Data

Another significant barrier in the development stage, on which significant progress has been made, is allowing access to patient data while maintaining patient confidentiality. Ideally data should be homogeneous such that systems can continually improve and be more easily shared between healthcare providers. If data between facets of the NHS is homogeneous this permits interoperability and relatively simple application of the same technology elsewhere. The technology may also be reprogrammed to meet new and emerging needs or integrate with a growing pool of data. Ultimately, if interoperability within a health system is achieved then this ensures that the technology is self-referential. In this way, as more data is acquired the effectiveness of the system increases and network effects begin to emerge, such that once an early adopter hospital takes on the technology the marginal benefit for each successive hospital now increases since the pool of data used to refine the technology continues to grow [92, 95].

Since 2019, NHSX has hosted efforts to establish a framework for managing NHS Health Data [96]. This has been complimented by efforts by Health Data Research UK to build a world-leading health research database for innovation [97]. However, the active work by NHS Digital and NHSX to improve the interoperability of NHS systems still has a long way to go, and this remains a major barrier to innovation in the UK [86].

One example of a successful innovation in the field of clinical data within the NHS is the digitalisation the ‘red book’ maternity record for parents to keep a record of their child’s development, including their immunisation records and growth [98]. This has the potential to be particularly useful in the long-term, with the NHS Long Term Plan noting that this will help children start their lives with a digital Personal Health Record (PHR) [48].

### Challenges to Invention: Accessing Funding and Support

The 2006 Cooksey review identified two translational gaps in the pipeline from laboratory to bedside [19]; firstly, translating basic research into new products, and secondly new products into clinical practice. Most innovation pathways focus on the latter gap, or later on in the first gap (i.e. from nascent product to tested prototype). Nevertheless, access to funding in the early stages of innovation is an issue, one which there is some support for. Much of this support lies outside traditional innovation pathways, such as venture capital and angel investors. Additionally, grant funding is available from university, government, private firm and EU sources [20].

The innovation pathways themselves provide help by guiding companies and individuals to funding, similar to how they provide access to testing. NIHR funding schemes also provide some direct funding, or links to investment, such as InnovateUK. This being said, a recent review highlighted the concern from NHS managers that austerity measures limit the amount of initial funding available from AHSNs [99], and that significant capital needs to be invested in the first place to access the benefits of AHSNs (i.e. the network of academia, clinicians and industry). Therefore, early funding from NHS sources remains a major area for improvement. The NIHR AI fund may provide a model for how NHS or Department of Health and Social Care funding can be used to encourage early-stage innovation in the future. We suggest that coordinating this should be a major role for the AAC going forward, through their Innovation Portal [100].

### Challenges to Invention: Encouraging Innovation

An emerging theme of the innovation pathways is the utility of the AHSN network as an asset for the coordination and guiding of innovators through the highly complex NHS innovation landscape, from the earliest invention stage right through to implementation. However, there is an even earlier stage of innovation that exists that is often ignored, and lies outside the remit of the AHSNs; encouraging clinicians (rather than more traditional academic/industry) to innovate. An example of this is the AAC’s Clinical Entrepreneur Training Programme (see above) [37], which takes on a cohort of NHS clinicians and teaches them skills which will aid innovation (similar to the Healthcare Leadership Academy [79]). Alternatively, the NHS Innovation Accelerator aims to seed innovations specific to their themed calls (see above), giving clinicians a chance to develop innovations in areas most needed by the NHS. Applicants submit their credentials and potential ideas within these calls, rather than the more informal process available for other innovations. This is another example of the ‘demand signalling’ necessary to gain innovations that are most useful to the NHS.

### Concluding Remarks

Our research indicates that there is a wide array of support available to innovators within the UK healthcare system. However, these support pathways are fragmented and there is notable redundancy among them. We suggest that expanding and clearly defining the leadership role of the AAC in the innovation landscape would go some of the way to removing this fragmentation.

As we tend towards the development of digital innovations, software and artificial intelligence, data homogenisation and interoperability will become increasingly important. Although already a goal of NHSX, we highlight this as a primary target for the NHS, as it could remove many of the current barriers to implementing innovations across regional and local NHS services. We also highlight the need for support for innovators at the earliest stages of the innovation process, invention. A lack of seed funding or support for those with a promising idea can act as a barrier to entry even if later stages of innovation development are well-supported.

While we have been able to provide a broad overview of the innovation landscape in the NHS, our research has been limited by the lack of primary literature on this topic, including few papers with input from innovators and clinicians. There is therefore a pressing need for a high-quality, independently funded review of the innovation landscape, as many of the above pathways have received no review, or if they have, they tended to be reviewed either by their own members, or by reviewers funded by the organisation under scrutiny. This could lead to bias in reporting and evaluation, whether real or perceived [101]. Such a review would ideally include a survey of relevant stakeholders to gain an insight into practical challenges faced by those who work with the pathways.

Given the broad range of innovation pathways within and around the NHS, it is no surprise that the NHS is a leading force in driving healthcare innovation globally. There is an array of support available for clinical innovators as well as for others seeking to improve patient care. Indeed, there are also opportunities for patients themselves to drive innovation and many of the pathways strongly encourage patient and public involvement. The pace at which these innovation pathways are growing offers an exciting prospect for the future of healthcare delivery in the United Kingdom.

## Abbreviations

AAC: Accelerated Access Collaborative
AHSC: Academic Health Science Centre
AHSN: Academic Health Science Network
AI: Artificial Intelligence
ARC: Applied Research Collaboration
CCG: Clinical Commissioning Group
CLAHRC: Collaborations for Leadership in Applied Health Research and Care
EU: European Union
HTA: Health Technology Assessment
i4i: Invention for Innovation
NHS: National Health Service
NHSx: UK joint organisation for digital, data and technology
NICE: National Institute for Health and Care Excellence
NIHR: National Institute for Health Research
MHRA: Medicines and Healthcare Products Regulatory Agency
GDP: Gross Domestic Product
PDA: Product Development Award
UK: United Kingdom
UKRI: United Kingdom Research and Innovation
RMOC: Regional Medicines Optimisation Committees
SBRI: Small Business Research Initiative
